# Prenatal exposure to the mineralocorticoid receptor antagonist spironolactone disrupts hippocampal area CA2 connectivity and alters behavior in mice

**DOI:** 10.1038/s41386-024-01971-7

**Published:** 2024-09-05

**Authors:** Stephanie M. Jones, Sarah Jo Sleiman, Katharine E. McCann, Alan K. Jarmusch, Georgia M. Alexander, Serena M. Dudek

**Affiliations:** 1 Neurobiology Laboratory, National Institute of Environmental Health Sciences, Division of Intramural Research, National Institute of Health, Research Triangle Park, NC 27709 USA; 2 Immunity, Inflammation, and Disease Laboratory, Division of Intramural Research, National Institute of Environmental Health Sciences, National Institutes of Health, Research Triangle Park, NC 27709 USA; 3 Present Address: Neuroscience Curriculum, University of North Carolina, Chapel Hill, NC 27599 USA; 4 Present Address: School of Psychology, Georgia Institute of Technology, Atlanta, GA 30332 USA

**Keywords:** Axon and dendritic guidance, Stress and resilience

## Abstract

In the brain, the hippocampus is enriched with mineralocorticoid receptors (MR; *Nr3c2*), a ligand-dependent transcription factor stimulated by the stress hormone corticosterone in rodents. Recently, we discovered that MR is required for the acquisition and maintenance of many features of mouse area CA2 neurons. Notably, we observed that immunofluorescence for the vesicular glutamate transporter 2 (vGluT2), likely representing afferents from the supramammillary nucleus (SuM), was disrupted in the embryonic, but not postnatal, MR knockout mouse CA2. To test whether pharmacological perturbation of MR activity *in utero* similarly disrupts CA2 connectivity, we implanted slow-release pellets containing the MR antagonist spironolactone in mouse dams during mid-gestation. After confirming that at least one likely active metabolite crossed from the dams’ serum into the embryonic brains, we found that spironolactone treatment caused a significant reduction of CA2 axon fluorescence intensity in the CA1 *stratum oriens*, where CA2 axons preferentially project, and that vGluT2 staining was significantly decreased in both CA2 and dentate gyrus in spironolactone-treated animals. We also found that spironolactone-treated animals exhibited increased reactivity to novel objects, an effect similar to what is seen with embryonic or postnatal CA2-targeted MR knockout. However, we found no difference in preference for social novelty between the treatment groups. We infer these results to suggest that persistent or more severe disruptions in MR function may be required to interfere with this type of social behavior. These findings do indicate, though, that developmental disruption in MR signaling can have persistent effects on hippocampal circuitry and behavior.

## Introduction

Brain corticosteroid receptors, including the glucocorticoid and mineralocorticoid receptors (GRs and MRs), are important regulators of the hypothalamic-pituitary-adrenal (HPA) axis and function in learning, memory, and behavioral adaptations to stress [1–4]. The hippocampus expresses both GR and MR, where they operate as ligand-inducible transcription factors upon binding endogenous stress hormones cortisol or corticosterone (“cort”) [5]. Interestingly, staining for MR is highly enriched in area CA2, a part of the hippocampus with a peculiar resistance to long-term potentiation (LTP) [6–10]. Recent studies have demonstrated a necessity for CA2 neurons in aggression, social memory, and novelty detection [11–15], and MRs are required for an animal’s preference towards social novelty and reaction to contextual novelty [16–18]. Importantly, variations of the gene encoding MRs (*NR3C2*) have been implicated in developmental and psychiatric disorders such as autism spectrum disorder and depression [19, 20].

Molecular and functional maturation of CA2 occurs early in postnatal life in mice, around postnatal day 12 [21–23]. However, expression of MR, but apparently not GR, is concentrated in hippocampal pyramidal cells beginning in the embryo, and MR expression both precedes and controls the expression of most known CA2 markers [16, 21, 24]. We have previously shown that MR knockout caused striking effects on CA2’s ‘synaptic plasticity phenotype’ and downregulation of genes enriched in CA2 [16]. Further, embryonic (Nestin-Cre; MR fl/fl), but not postnatal (Amigo2-Cre; MR fl/fl), deletion of MR decreased staining for vGluT2, which has been shown to label axon terminals from the SuM neurons [25]. Input from the SuM appears to be critical to CA2’s function in social behavior [14, 16, 26, 27]. Despite what is known about the actions of MRs in regulating CA2 genes from our studies using knockout mice and pharmacological manipulation in adult animals, little is known about the effects of MR disruption that is limited to early development. We hypothesized that disturbing MR function during early development may impair the establishment of CA2 connectivity, and with it, its role in behavior.

Prenatal pharmacological manipulation of hormone signaling has previously been used in several animal models, and these studies report persistent effects on differentiation, development and function of target organs long after exposure ceases [28–30]. In this study, we used a similar approach to manipulate stress hormone signaling in the brain prenatally. Because knockout of genes using the Cre-LoxP recombination system in mice permanently excises the target gene, persisting throughout the life of the animal [31], and we aimed to limit MR signaling disruption to prenatal development, we took a pharmacological approach. We asked whether *in utero* exposure to the MR antagonist spironolactone (spiro) could disrupt the acquisition of CA2 molecular identity, connectivity, and/or CA2-dependent behaviors in adults. We found that although spiro-treated animals showed no change in some CA2 markers in adults, both CA2 neuron input (from the SuM) and output (to the CA1) connectivity were disrupted. Additionally, we found an increase in the animals’ reactivity to contextual novelty, similar to what has been shown in the MR conditional knockout mice [16–18]. These findings support the idea that MRs shape CA2 development and adult behavior. Furthermore, they illustrate a period of prenatal life when CA2 neuron structure is vulnerable to disruption by a pharmacological agent.

## Materials and methods

### Timed pregnancies & pellet implantation

Male C57BL/6J mice and female animals expressing green fluorescent protein under the *Amigo2* promoter (Amigo-2 GFP; Gene Expression Nervous System Atlas, Amigo2-EGFP; Tg(Amigo2-EGFP)LW244Gsat/Mmcd) were paired for timed mating. Dams were monitored for copulation plugs and separated from sires after being paired for 48 h. Dams were also weighed for several days to confirm progression of pregnancy (≥3 g over base-weight). Using the presence of a copulation plug as the first gestational day, surgeries were performed on gestational day 12 (+/−1 day). Dams were anesthetized using 2.5% isoflurane and subcutaneously implanted with 21-day release pellets containing either spiro or vehicle (15 mg/pellet, spiro, Cat. No. M-161, vehicle, Cat. No. C-111 Innovative Research of America, Sarasota, FL, USA) alongside a no-surgery control group. The 15-mg spiro pellets follow zero-order kinetics, yielding a constant dose over the 21-days. The spiro pellets are predicted to release ~0.7 mg spiro/day, yielding an approximate dose of 20 mg/kg/day. Vehicle pellets were a biodegradable matrix of the carrier-binder consisting of cholesterol, lactose, celluloses, phosphates, and stearates. The same matrix was used for the spiro pellets with the drug included in the matrix for a slow, continuous release.

Dams were maintained on a breeding chow for the duration of gestation (LabDiet, Cat. No. 5KON). Although midgestational administration of a 21-day release pellet could possibly expose pups postnatally, we consider the dosing “prenatal” due to the poor excretion of spiro into breastmilk [32]. Pups were weaned at approximately P21 and offspring were housed with same-sex littermates until P60 (+/−3) when behavioral assessments were performed, after which animals were sacrificed for immunohistochemical analysis. All animal protocols were approved by the National Institute of Environmental Health Sciences Animal Care and Use Committee and are in accordance with the National Institutes of Health guidelines for care and use of animals. Mice were housed on a 12 h:12 h light-dark cycle with same-sex littermates, with up to five animals per cage. Mice were provided water and food *ad libitum*. For all measures in adult subject offspring, we found no difference between males and females or between vehicle-treated and non-surgical controls, so we combined data from males and females within each treatment and combined vehicle-treated and non-surgical controls into a single control group.

### Tissue collection and HPLC-MS analysis

Dams and pups were sacrificed for serum or brain tissue (pups) collection on the day of parturition and subsequent analytical confirmation of spiro and its metabolites using liquid chromatography-mass spectrometry (LC-MS). Dams were anesthetized using sodium pentobarbital (50 mg/mL; >100 mg/kg intraperitoneal (IP) injection), killed by decapitation, and trunk blood captured into a 1.5 mL tube. Blood coagulated for 15–30 min at room temperature and spun for 10 min at 4500 rpm at 4 °C. The serum fraction was collected and stored at −20 °C until analysis. Pups were decapitated and brains collected into a prechilled 1.5 mL tube before storage at −70 °C.

The following stock reagents were created into standard solutions to confirm accurate mass and retention time of analytes: spiro (Cayman Chemical Company; 9000324), canrenone (“can”; Cayman Chemical Company; 21307), 7α-thiomethylspironolactone (“7-alpha”; Santa Cruz; sc-207187), and 6β-Hydroxy-7α-thiomethylspironolactone (“6-beta”; Santa Cruz; sc-210569). Spiro and its metabolites do not ionize efficiently in MS instruments, and so stock reagents and samples were subjected to a pretreatment with a derivatizing agent, Girard’s reagent P(GP), to increase signal responses [33].

In brief, pup brains were bead homogenized in HPLC-grade water (200 mg tissue/mL water) in an Omni Bead Ruptor Elite. Serum samples from dams (*n* = 4 dams/group; non-surgical and vehicle mixed for control group) and brain homogenates of pup brains from one litter of each spiro and non-surgical groups (*n* = 3 pups/litter/experimental group) were processed in parallel. Proteins were removed using methanol precipitation and centrifugation and the supernatant subjected to Girard P derivatization [34]. Samples were analyzed using an ultra-high performance liquid chromatograph (Vanquish^TM^ Horizon UHPLC, Thermo Scientific) coupled to a high-resolution mass spectrometer (Orbitrap Fusion^TM^ Tribrid, Thermo Scientific). Data were extracted using FreeStyle from ThermoFisher Scientific, and peaks were integrated using the default setting for the monoisotopic mass of each derivatized analyte with 5 ppm mass error. All values for integrated peaks of spiro and its metabolites were “0”, or not detected, for tissues collected from non-dosed animals. Thus, to facilitate comparisons, all integrated peak areas were log_2_(x + 1) transformed before statistical analysis. Metabolites in dam serum and pup brains were compared using a two-way ANOVA with multiple comparisons.

### RNA in situ hybridization

A wildtype C57BL/6J mouse was sacrificed on the day of birth (P0) for RNA in situ hybridization (RNAscope). The mouse was sacrificed via rapid decapitation and the brain was harvested in RNAse-free conditions. The brain was frozen in Tissue Plus® O.C.T Compound (Fisher Scientific, Hampton, NH) and stored at −70 °C until cryo-sectioning at 10 μm. In situ hybridization was performed according to the manufacturer’s protocol for the RNAscope Fluorescent Multiplex kit (Advanced Cell Diagnostics, Hayward, CA) using Mm-*Nr3c2*-E5E6-C3 (Cat#456321-C3) and coverslipped with Vectashield Hardset Mounting Medium containing a DAPI nuclear counterstain (Vector, Burlingame, CA). Stained sections were imaged on a Zeiss LSM 880 inverted confocal microscope.

### Behavior

All behavioral experiments were conducted during the light cycle. Animals were brought to the behavior room and habituated for 30 min prior to each behavioral assay. Before and after each animal was placed into and removed from the respective behavioral arenas, a cleaning solution was used to thoroughly sanitize the floors, walls, and objects or cups of testing areas. Animals assessed for anxiety-like behaviors (Open Field) and reactivity to novel objects included 32 controls (18 males from 8 litters and 14 females from 5 separate litters) and 20 spiro-treated (10 males from 4 litters and 10 females from 3 separate litters). No significant differences were found between sexes or between litters with a treatment group (*p* > 0.05, two-way ANOVAs with Sidak’s multiple comparisons tests) so males and females from all litters were combined within each treatment group. Animals were placed in a novel, empty arena to freely explore for 5 min before returning to their home cage. Noldus Ethovision was used to track the animals’ locomotion in this novel environment, including measurements of distance traveled, velocity, and time spent (s) in the center of the arena. On the following day, animals were re-introduced to the same arena to explore for 10 min to serve as a habituation period. On the third day, novel object reactivity was assessed by placing two identical novel objects (glass vials) along opposing walls of the same arena. Animals were placed in the arena and allowed to investigate the novel objects for 10 min before being returned to their home cage. Noldus Ethovision was used to record and track the movement of the mice and the time spent interacting with the objects in their respective zones (“interaction zones”). To calculate novel object reactivity for each animal, the times spent in each interaction zone were summed. All comparisons between collapsed-control and spiro exposed were performed using two-tailed t-tests.

Animals tested for sociability and social novelty preference included 26 controls (12 males from 6 litters and 14 females from 5 separate litters) and 14 spiro-treated mice (8 males from 3 litters and 6 females from 2 separate litters). No significant differences were found between sexes or between litters within a treatment group (*p* > 0.05, two-way ANOVAs with Sidak’s multiple comparisons tests), so males and females from all litters were combined within each treatment group. The social assays were performed using a three-chamber arena with doorways between each chamber. The left and right chambers contained mesh cups in their respective extreme corners, large enough to hold a conspecific. The subject animal was placed in the center chamber at the start of the first trial and permitted to investigate the three chambers freely for 10 min. At the end of the 10 min, the animal was ushered back into the center chamber and the doorways closed while a novel sex- and age-matched conspecific was added to one of the mesh cups. The second trial began with re-opening the doorways and the subject animal was allowed to explore again for 10 min to habituate to the novel animal. At the end of the second trial, the subject animal was ushered back into the center chamber and the doorways closed as a different (now novel) conspecific (sex- and age-matched from a different litter than the first stimulus) was placed in the mesh cup. The third trial began with re-opening the doorways and the animal allowed to explore the 3 chambers again for 10 min. Placement (left or right chamber) of the novel conspecifics for the second and third trials were randomly assigned to counterbalance across subject animals. Between each subject animal, the arena and cups were thoroughly cleaned. Neuromotive software (Blackrock Neurotech) was used to record videos of each trial, which were later imported into the Noldus Ethovision software for the tracking and analysis of the subject mouse during trials two and three. Social discrimination was calculated as the ratio of time the animal spent in the interaction zone around the novel conspecific divided by the time spent in the interaction zone about the familiarized conspecific (“discrimination ratio”) for each animal. Comparison between controls and spiro-exposed animals were performed using a two-tailed t-test.

### Immunofluorescence imaging and analysis

After behavioral assessment, a subset of litters was arbitrarily selected for immunostaining and tissue processing, and only those animals that were GFP+ used, including 10 (9 male, 1 female) control animals (from three vehicle litters and one non-surgical litter, no significant litter effects detected, *p* > 0.05) and 6 (4 male, 2 female) spiro-treated animals (from two litters, no significant litter effects detected, *p* > 0.05, two-way ANOVAs with Sidak’s multiple comparisons tests). GFP-expressing animals were sacrificed to facilitate localization of CA2 neurons during immunostaining. Staining, imaging, and analysis were performed with the experimenter blinded to condition. Briefly, mice were anesthetized with Fatal Plus (sodium pentobarbital, 50 mg/mL; > 100 mg/kg IP injection) and transcardially perfused with phosphate-buffered saline (PBS) followed by 4% paraformaldehyde. Brains were harvested and post-fixed in 4% paraformaldehyde at 4 °C for 24 h. Brains were sectioned coronally on a vibratome at 40 μm and stored at 4 °C in PBS with 0.02% sodium azide until future processing. For immunofluorescent staining for CA2 markers (NECAB2, PCP4, and MR), sections were rinsed with PBS, permeabilized with in 0.3% TritonX PBS, and blocked for 1 h with 5% normal goat serum in 0.3% TritonX PBS, prior to overnight incubation at 4 °C in the following primary antibodies in 5% normal goat serum in 0.3% TritonX PBS (see supplemental material for details): rabbit anti-PCP4, rabbit anti-NECAB2, mouse anti-MR. Sections were then washed 2 ×10 min in TritonX PBS and then incubated for 2 hours with the appropriate secondary antibodies conjugated to fluorescent probes. Tissue was rinsed 2 ×10 min with 0.1% Triton X in PBS and washed with PBS before mounting in Vectashield Hardset Mounting Medium with a DAPI nuclear counterstain (Vector, Burlingame, CA).

For visualization of CA2 axons and afferents presumably from SuM, we immunostained sections for GFP signal and vGluT2 protein, respectively. The staining procedure was identical to above, with the following modifications: 0.1% TritonX PBS was used instead of 0.3% for permeabilization; 3% normal goat serum in 0.2% TritonX PBS was used for blocking overnight; primary antibodies (guinea pig anti-vGluT2 and chicken anti-GFP) were diluted in 3% normal goat serum PBS and were incubated for 48 h. Sections were washed 2 ×15 min with 0.2% TritonX PBS before incubation in fluorescent secondary antibodies with 3% normal goat serum in PBS: AlexaFluor 568 goat anti-guinea pig and AlexaFluor goat anti-chicken 488 for 5 h at room temperature. Sections were rinsed 2 ×15 min with 0.2% TritonX PBS and 1x PBS for 10 min before mounting with Vectashield Hardset Mounting Medium with a DAPI nuclear counterstain (Vector, Burlingame, CA). Tissue across treatment groups were processed concurrently, in the same reaction conditions, for each staining experiment to facilitate comparisons.

All images for quantification were acquired on a Zeiss AxioObserver.Z1 microscope by an experimenter blinded to treatment. Quantifications were performed in ImageJ (1.49 v) using images collected between −1.7 and −1.9 mm posterior to bregma. For quantification of CA2-localized stains (PCP4, NECAB2, MR, GFP, vGluT2, somatic GFP), a free-form region-of-interest was circumscribed around marker staining in the GFP channel for measurement in other analysis channels. For these, background signal in fluorescence images were subtracted using an area outside of the hippocampal pyramidal layer. For quantification of vGluT2 immunofluorescence in the DG, a 250 ×250 pixel square region-of-interest was created and positioned over the outer granule cell layer, localized over vGluT2 staining, of the granule cell layer. Immunofluorescence values were averaged from two to three sections for each animal. Comparisons between control and spiro-exposed mice were completed using a two-tailed t-tests. Presented images are brightness/contrast adjusted equally across treatment groups for each stain.

For analysis of CA2 axonal projections, fluorescence signal from axonal structures was evaluated in treated and control Amigo2-GFP offspring. A linear region-of-interest was created across hippocampal cell layers (SO, SP, SR, SLM) starting at the bottom of alveus and ending at the border of SLM and the suprapyramidal blade of the DG. This linear region-of-interest was placed parallel to the midline and perpendicular to the pyramidal cell layer. Similar coronal planes were used for each animal (~1.82 mm posterior to bregma). Fluorescence intensity was extracted at every pixel distance along this line, binned at 10 μm, and plotted as a signal intensity plot. Maximum fluorescence intensity values of the SO and SR regions were extracted from each animal across groups and compared using two-way, repeated measures ANOVA with post hoc comparisons. GFP + CA2 neurons were quantified in the same sections as with axon targeting analysis and manually scored using Imaris (Oxford Instruments plc, Abington UK, v10.1).

## Results

### Spiro metabolites can be detected in dam serum and neonatal brain tissue

**Fig. 1:**
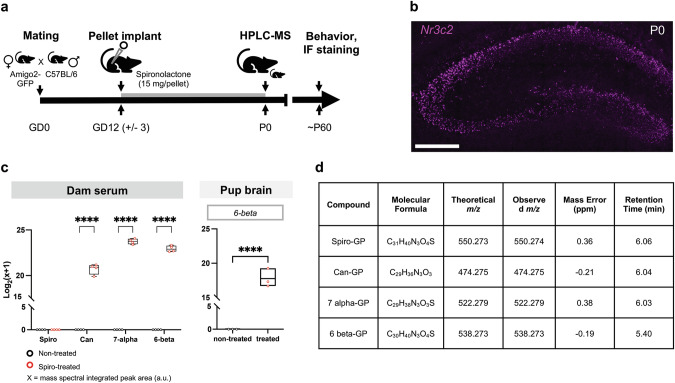
Spiro metabolites are detected in treated dams and pups. **a** On the first day of pregnancy (gestational day 0; GD0), each Amigo2-GFP dam was paired with a male C57BL/6J mouse. Pregnancy was confirmed by the presence of a copulation plug after pairing. Spiro pellets (15 mg/pellet) were implanted in pregnant dams at gestational day 12 (+/−1; GD12). High-performance liquid chromatography-mass spectrometry (HPLCMS) was used to confirm the presence of spiro and metabolites in a subset of dams and pups collected on the day of birth (first postnatal day; P0). Other litters were allowed to age until P60 for behavioral assays prior to immunofluorescent studies. **b** RNA in situ hybridization of the gene encoding MR, *Nr3c2* (magenta), in a murine hippocampus at P0. Scale bar is 200 µm. **c** Log_2_(x + 1) transformed peak integrated values (arbitrary units; a.u.) of spiro and metabolites in serum and brain homogenates of treated (red circles) and control (black circles) subjects. Displayed analytes include spiro and metabolites canrenone (can), 7α-Thiomethylspironolactone (7-alpha), and 6-beta-7-alpha-thiomethylspironolactone (6-beta). Two-way ANOVA analysis revealed that there was a main effect of treatment on transformed integrated peak values (two-way, ANOVA, *F*(1,6) = 16363, *p* < 0.0001). Post hoc analysis revealed that integrated areas differed between control and treatment groups for can, 7-alpha, and 6-beta (Šídák multiple comparisons test, *p* < 0.0001 for all except for spiro, which was not detected). One metabolite of spiro, 6-beta, was detected in pup brains of a spiro-treated litter on the day of birth (one-tailed, unpaired t-test, *t*(4) = 22.94, *p* < 0.0001). Data are presented in box and whisker (min to max, line at median) and floating bar (min to max, line at mean) plots in Prism for serum and brain homogenate data, respectively. **d** Table showing mass spectral measurements of spiro and metabolites measured by LC-MS.

We previously demonstrated that treatment with spiro, an MR antagonist, delivered via subcutaneously implanted slow-release pellets resulted in decreased expression of the CA2 marker RGS14 in adult animals (McCann et al. [16]). This finding suggests that spiro – or a metabolite- in a pellet formulation can penetrate brain tissue and modulate protein expression. Here, we implanted spiro pellets in gestating mice with the aim of inhibiting MRs in the prenatal embryo. To ensure that prenatal spiro treatment was sufficient to reach systemic circulation in a pregnant animal and cross the placental barrier to reach brain tissue of offspring, we performed LC-MS of dam serum and pup brain homogenates in control and treated groups (Fig. 1). Spiro was not detected in serum or brain tissue samples, likely owing to the rapid metabolism in vivo of the prodrug [35]. Instead, we detected canrenone (“can”), 7α-thiomethylspironolactone (“7-alpha”), and 6β-hydroxy-7α-thiomethylspironolactone (“6-beta”), major metabolites of spiro in the serum of treated dams [35–40]. The lipophilic nature of spiro and its metabolites is thought to permit their passage across the blood-brain and placental barriers [41]. As such, we did detect 6-beta in treated pup brains. These data indicate that the drug delivered by pellet implant did in fact reach circulation in the dam and that at least one metabolite crossed the placenta into brains of the pups.

### CA2 innervation of dorsal CA1 is disrupted in treated animals

**Fig. 2:**
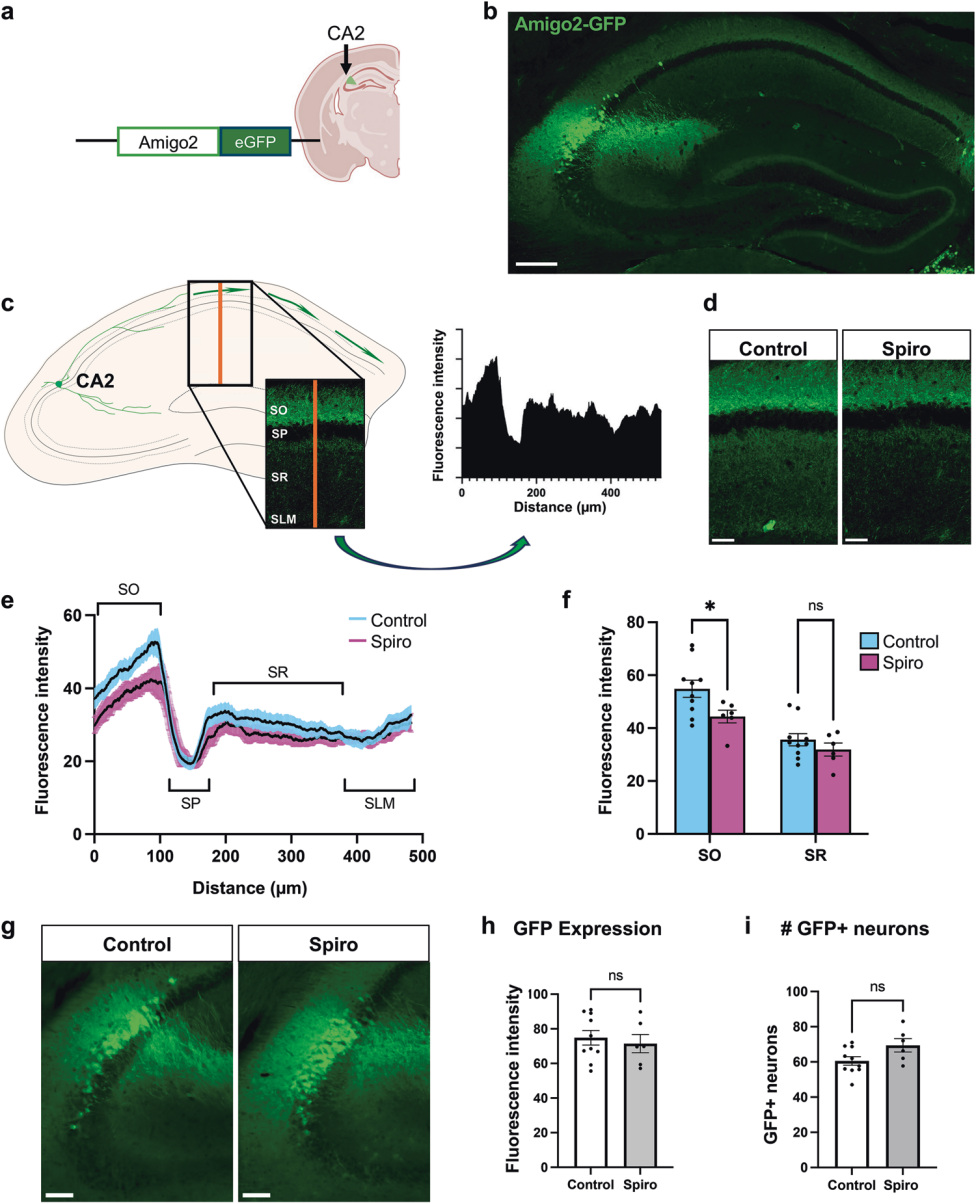
CA2 innervation of dorsal CA1 is decreased following prenatal spiro. **a** Schematic of green fluorescent protein (GFP) expression being driven by the *Amigo2* promoter. Amigo2 and GFP are selectively expressed in CA2 of this mouse strain. Image created in Biorender. **b** Representative image of GFP expression in the murine hippocampus. **c** To assay CA2 axonal profile, coronal hippocampal sections were immunostained for GFP to enhance endogenous fluorescence. A 10-pixel width linear region-of-interest (ROI) was created through CA1 to capture CA2-axonal GFP signal through the *stratum oriens* (SO), *stratum pyramidale* [65], *stratum radiatum* (SR), and *stratum lacunosum-moleculare* (SLM) cell layers. Fluorescence intensity values along the line were plotted for the creation of line intensity plot for each animal. Of note, CA2 axonal signal is most dense in SO and SR layers. **d** Representative images of CA2 axonal signal in CA1 of adult animals from control (left) and spiro-treated litters (right). **e** Averaged line intensity profile plots for control and spiro-exposed animals. Lines are displayed with cyan and magenta SEM error bars for control and spiro litters, respectively. Control and spiro groups seemed to differ in GFP expression in SO and SR. **f** The maximum fluorescence intensity value from the SO and SR linear regions were extracted from each animal. We found no main effect for treatment (two-way, rmANOVA, *F*(1,14) = 3.239, *p* = 0.0935), but there was a significant interaction between layer and treatment (two-way, rmANOVA, *F*(1,14) = 8.842, *p* = 0.0101). Post hoc analysis revealed that maximum fluorescence intensity value differed in SO between control and spiro (Šídák multiple comparisons test, *t*(28) = 2.555, *p* = 0.0324), but not in SR (Šídák multiple comparisons test, *t*(28) = 0.9032, *p* = 0.6083). **g** Representative images of GFP immunofluorescence in CA2 of adult animals from control (left panel) and spiro-treated litters (right panel). **h** Adult, background-subtracted GFP immunofluorescence also did not differ (two-tailed, unpaired t-test *t*(14) = 0.4979, *p* = 0.6263). **i** Similarly, the number of GFP+ cells did not differ across groups (two-tailed, unpaired t-test *t*(14) = 2.075, *p* = 0.0569). Scale bars are: **b** 200 µm; **d** 50 µm; **g** 100 µm. Image created with BioRender.com.

CA2 neuron axons project to ipsilateral dorsal CA1, primarily targeting the *stratum oriens* (SO) layer, and to a lesser extent targeting *stratum radiatum* (SR; [42–44]). We asked whether MR blockade during development impacts the patterning of CA2 axon projections in CA1, which likely develop during the time that animals were exposed to spiro [45, 46]. We used dams expressing green fluorescent protein (GFP) under control of the *Amigo2* promoter (Amigo2-GFP), so treated pups also expressed GFP in CA2 pyramidal neurons, enabling visualization of their axons (Fig. 2a, b). To measure GFP fluorescence intensity of CA2 axons in dorsal CA1, we created a linear region-of-interest across the CA1 layers, SO*, stratum pyramidale* (SP), SR, and *stratum lacunosum-moleculare* (SLM) (Fig. 2c). We found that the axonal GFP signal profile differed between the treatment groups (Fig. 2d–f). GFP fluorescence was significantly decreased in SO of spiro-treated animals than controls, although no difference was detected in SR. To determine whether the decreased axonal GFP signal was simply a result of decreased GFP expression, we measured the fluorescence intensity in the CA2 pyramidal cell body layer between treatment groups (Fig. 2g, h). We additionally measured the number of GFP-expressing cells in sections and found no difference in cell number between treatment groups (Fig. 2i). Therefore, the decreased axonal GFP fluorescence in CA1 is unlikely to reflect a decrease in CA2 cell number or GFP expression, supporting the conclusion that prenatal spiro treatment perturbed CA2 axon number or guidance in dorsal CA1. Image created with BioRender.com.

### Prenatal exposure to spiro does not change adult expression of CA2 markers

**Fig. 3:**
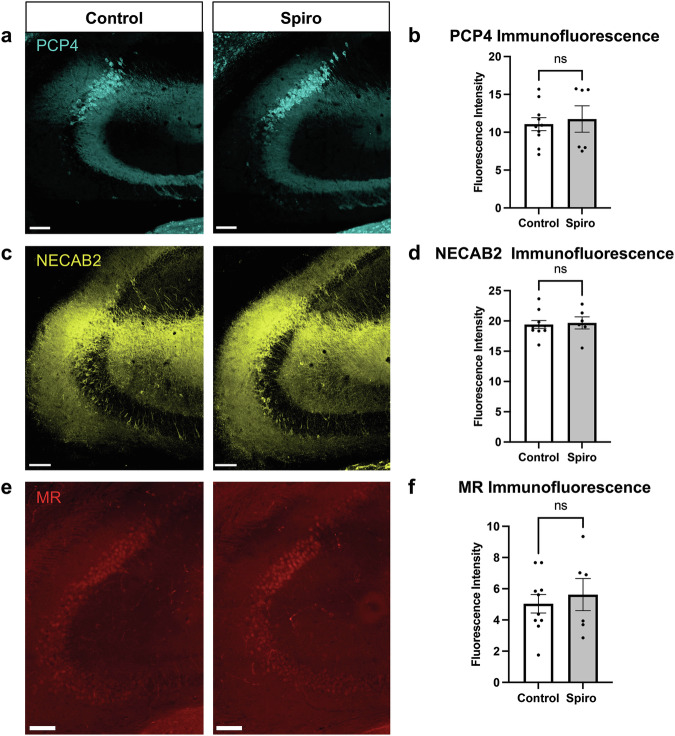
CA2 marker expression is unaffected by prenatal spiro exposure. **a** Representative images of PCP4 immunofluorescence in CA2 of adult animals from control (left panel) and spiro-treated litters (right panel). Scale bars are 100 μm. **b** Adult PCP4 expression in CA2 did not differ in offspring of control and treated litters (two-tailed, unpaired t-test, *t*(14) = 0.3903, *p* = 0.7022). **c** Representative images of NECAB2 immunofluorescence in CA2 of adult animals from control (left panel) and spiro-treated litters (right panel). Scale bars are 100 μm. **d** Adult NECAB2 expression in CA2 did not differ in offspring of control and treated litters (two-tailed, unpaired t-test, *t*(14) = 0.2339, *p* = 0.8185). **e** Representative images of MR immunofluorescence in CA2 of adult animals. **f** MR expression levels did not differ between control (left panel) and spiro-treated litters (right panel) (two-tailed, unpaired t-tests, *t*(14) = 0.5362, *p* = 0.6002). All scale bars are 100 µm.

CA2 cells express a unique complement of genes [47, 48], which is maintained by the cell-autonomous expression of MRs [16]. We proposed that MR may be a “terminal selector” transcription factor that functions principally to specify CA2 cell fate in early development of the hippocampus [16, 49, 50]. Therefore, we next asked whether prenatal MR blockade would be sufficient to change CA2 molecular identity in adulthood. Using immunofluorescence, we measured the protein expression of MR and two MR-dependent CA2 markers, PCP4 and NECAB2, in the CA2 pyramidal cell layer of adult animals that were prenatally exposed to spiro (Fig. 3). We found no difference in MR, PCP4, or NECAB2 immunofluorescence between spiro-treated animals and controls. Therefore, prenatal MR blockade does not cause any lasting expression of some CA2 protein markers as measured in adulthood.

### Prenatal spiro treatment disrupts hippocampal input connectivity

**Fig. 4:**
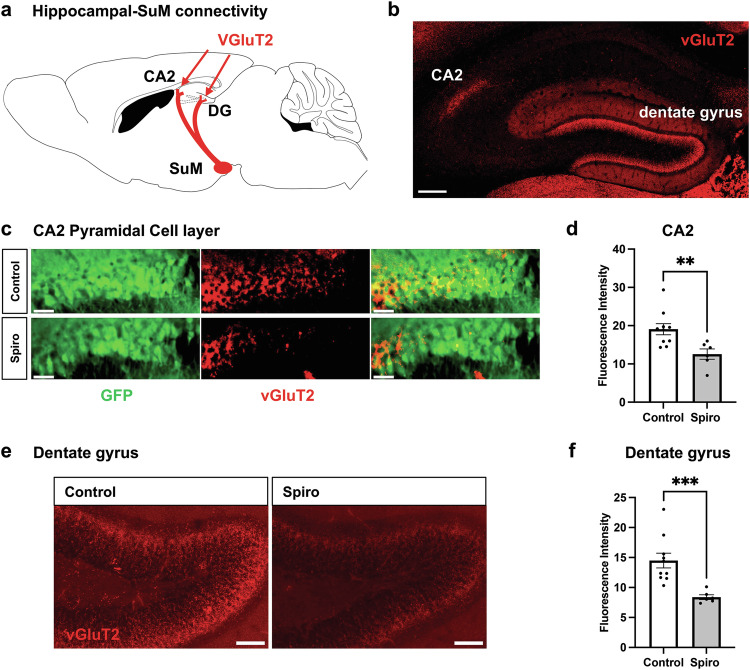
Prenatal spiro alters presumed SuM input into CA2 and the dentate gyrus. **a** Schematic representation of known SuM projections to CA2 and the DG of the hippocampus. SuM afferents are approximated by vGluT2 immunostaining in these regions, shown in an example in **b**. **c** Representative images of vGluT2 staining (red) in CA2 of control (top) and spiro-treated (bottom) mice. **d** Prenatal exposure to spiro led to decreased  vGluT2 immunostaining in CA2, suggestive of decreased SuM input into this region (two-tailed, unpaired t-test, *t*(14)  =  3.040, *p*  =  0.0088). **e** Representative images of vGluT2 (red) staining in the DG of control (top) and spiro-treated mice. **f** Spiro exposure led to decreased vGluT2 immunostaining in the DG, also suggestive of decreased SuM input into this region (two-tailed, unpaired t-test with Welch’s correction, *t*(10.78)  =  4.721, *p*  =  0.0007). Scale bars are: **b** 200 µm; **c** and **e** 50 µm.

Previously, we reported that in mice with MR deleted prenatally, using a Nestin-Cre line, staining for vGluT2 in CA2, which most likely labels axon terminals from SuM, was significantly decreased (Fig. 4a, b) [16, 51]. However, in mice with MR deleted postnatally, using an Amigo2-Cre line, vGluT2 staining was not significantly decreased. Given this apparent dependence of vGluT2 positive innervation of CA2 on embryonic MR expression, we asked whether vGluT2 staining may similarly be disrupted in spiro-treated mice. As the SuM is known to project heavily to both CA2 and DG and its axons can be stained with vGluT2 antibodies, we measured immunofluorescence for vGluT2 in both CA2 and the DG of adult animals in animals prenatally treated with spiro (Fig. 4c–f). We found that vGluT2 immunofluorescence was significantly decreased in CA2 and DG in spiro-treated animals compared with controls. These data suggest that prenatal MR inhibition causes a lasting loss of presumed SuM axons in the hippocampus.

### Prenatal spiro exposure causes hyper-reactivity to novel objects

**Fig. 5:**
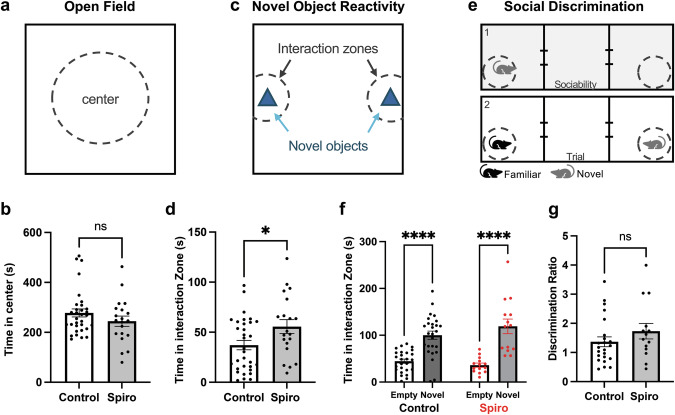
Prenatal spiro exposure leads to alterations in some CA2-dependent behaviors. **a** Mice were allowed to freely explore a novel open arena (Open Field) for a 10-minute period. Anxiety-like behavior was measured by determining the cumulative time spent in the center of the arena (less time in the center is interpreted as greater anxiety). **b** The total duration of time spent in center did not differ between control and exposed groups (two-tailed, unpaired t-test, *t*(50) = 1.274, *p* = 0.2085). **c** Animals were tested for novel object reactivity in a familiarized testing arena. **d** Animals exposed to spiro prenatally spent more time in the interaction zones about novel objects than controls (two-tailed, unpaired t-test, *t*(50) = 2.295, *p* = 0.0260). **e** For a 10-minute period, subject mice were allowed to explore a familiarized three-chamber arena containing one novel conspecific within a cup in one chamber and an empty cup in the opposing chamber, which served as a test for sociability (1). Mice were then introduced to a second novel conspecific contained in the opposing chamber (2). The time spent in the interaction zones over a 10-min duration about the stimuli mice was measured. **f** Mice in both treatment groups showed a significant preference for the cup with the novel animal over the empty cup. (main effect of chamber: F(1,76) = 59.72, *p* < 0.0001; each treatment group, *p* < 0.0001; two-way ANOVA with Sidak’s multiple comparisons test. **g** The ratio of time spent about the novel mouse to the time spent about the familiar mouse did not differ between control and spiro-treated groups (two-tailed, unpaired t-test, *t*(35) = 1.216, *p* = 0.2322).

SuM-CA2 and SuM-DG circuits are activated by social and contextual novelty, respectively [14], and we previously found that MR deletion, either brain-wide or CA2-targeted, results in behavioral hyper-reactivity to novel objects and a loss of preference for social novelty [4, 16, 18]. Therefore, we asked if mice exposed to spiro prenatally, and thus with a likely disruption of SuM input, would similarly show differences in response to novel social and object stimuli. We found that spiro-treated animals showed hyper-reactivity to novel objects, evidenced by an increased cumulative duration of time spent in interaction zones about the objects (Fig. 5c, d). We also tested social behavior in animals using the 3-chamber social assay. Similar to our findings with the conditional MR knockout animals, we found that animals in both treatment groups showed a significant preference for the cup with the novel animal over the empty cup (Fig. 5e, f). However, in contrast to data from conditional knockout mice, we did not detect a significant difference in preference for a novel animal over a familiar animal; prenatal spiro-treated animals did not differ significantly in the ratio of time spent with the novel vs familiar mice when compared to controls (Fig. 5e, f). We additionally tested spiro-treated animals and controls for measure anxiety-like tendencies in an open field assay. We found no difference in time spent in center (Fig. 5a, b), distance traveled, or velocity (two-tailed, unpaired t-tests, distance traveled, *p* = 0.6221, velocity, *p* = 0.6347; not shown) in the open field, suggesting no effect of treatment on anxiety-like behaviors. These data suggest that preference for social novelty differs from reactivity to novel objects in its (lack of) susceptibility to prenatal MR inhibition; postnatal disruption of MR function or lasting changes in gene expression may be required for some social behaviors like social memory.

## Discussion

Pyramidal neurons in CA2 appear to play an important role in social recognition memory, yet little is known about the functional significance of MR expression there. Of interest is the enrichment of MR expression in CA2 not only in adults, but also early in development [24]. We previously showed that MR knockout mediated by *Nestin* promoter-driven Cre recombinase disrupted the molecular and synaptic profile of CA2 pyramidal neurons [16]. *Nestin* is specifically expressed during development in neuroepithelial stem cells and, thus, *Nestin-*driven Cre expression is established in all neurons of the brain beginning in development at embryonic day 7.75 [52]. However, from this, it was unclear whether embryonic MR activity alone is sufficient to drive the changes we observed, given the irreversible nature of the Cre-driven excision of LoxP-flanked MR allele. Therefore, to disrupt MR function *in utero*, we used gestational treatment of dams with spiro to temporally-confine MR blockade. We confirmed the release of spiro from the subcutaneous pellet and presence of major spiro metabolites in both the dam serum and pup brain. As 6-beta was the only major metabolite detected in the pup brain, we suggest that 6-beta is responsible for the spiro-induced effects. Although spiro itself can bind androgen receptors (ARs) and progesterone receptors, albeit at a lower affinity to than binding to MR [36, 38], less is known about the efficacy and specificity of the metabolites like 6-beta [37]. That said, we think the observed effects of prenatal spiro were due to activity at MRs because we know of no evidence that ARs are expressed in the mouse hippocampus during the treatment window. To the contrary, it appears that AR expression does not increase to substantial levels until sometime after postnatal day 4 (P4), with some enrichment in CA2 by P14 (Allen Developing Mouse Brain Atlas). Future studies, however, will be required to determine the free concentrations of 6-beta in the embryonic brain as well as its binding and functional affinities at MRs (and ARs). Our findings reveal that prenatal MR inhibition is sufficient to alter CA2 circuitry, including inputs, likely arising from SuM, and outputs to dorsal CA1, as well as to change behavior in adulthood. We conclude that embryonic MR activity plays a fundamental role in establishing the connectivity and function of CA2 neurons that persists into adulthood. However, prenatal MR inhibition did not cause lasting disruption of a number of CA2 markers detected in adulthood, suggesting that the molecular fate of CA2 neurons was not disrupted by this treatment. That adult social behavior was *not* affected in the prenatal spiro treated animals, though, is inconsistent with findings from our previous study in which pre- or post-natal knockout of MR, which *was* sufficient to impair adult social behavior [16, 18]. Thus, a more prolonged MR blockade, or temporary blockade of MR function during a different critical time window may be required for impairment of preference for social novelty. Alternatively, homeostatic type mechanisms may be compensating at the synaptic level for the early loss of MR function.

The primary target of CA2 neurons within the hippocampus is the SO in dorsal CA1, as well as in the CA1 SO and SR at more caudal levels [44, 53]. Animals exposed to spiro embryonically showed reduced CA2 innervation of SO (in dorsal CA1), as measured by axonal expression of GFP arising from CA2 neurons. Neither CA2 cell number nor somatic GFP fluorescence were decreased, supporting our conclusion that it is the CA2 axons that were mistargeted or else failed to develop the normal number of axon collaterals. Because MR expression is required for expression of all tested CA2-enriched molecular markers [16], we suggest that MR may be upstream in the transcriptional control of CA2-unique guidance cues such as growth factors like NT3, that are diffusible to the extracellular environment [54]. Alternatively, MR may be important for expression of structural scaffolds like perineuronal nets, that could be responsible for the stabilization of SuM axons to these regions, as suggested previously [16].

The CA2 to dorsal CA1 subcircuit remains to be fully characterized for its functional consequences and role in behavior, although it does not appear to be required for social recognition memory [55], instead relying on ventral CA1 for CA2’s requirement in social memory^56^. We did not investigate CA2 axonal inputs to ventral CA1, so the contribution of that pathway to the normal social discrimination we observed in spiro-treated animals is to be determined. However, the CA2 to dorsal CA1 subcircuit has been implicated in novel object recognition [55], so the altered response to novel objects in our spiro-treated animals may be partially explained by the impaired CA2 innervation of dorsal CA1, which likely impaired CA2 to CA1 synaptic communication.

SuM input to each of CA2 and DG have been found to signal contextual and social novelty, respectively, in mice [14]. In their study, Chen et al. [14], reported that largely non-overlapping populations of SuM neurons were excited by either contextual or social novelty, although approximately one-third of SuM neurons responded to both stimuli. Retrograde labeling experiments similarly revealed largely non-overlapping SuM populations project to each region, although approximately one-quarter of neurons projected to both regions. We found that embryonic inhibition of MR had a pronounced impact on vGluT2 staining, which likely represents SuM terminals in both CA2 and DG. This finding suggests that embryonic MR activity, likely in CA2 and possibly in DG neurons, is required for appropriate establishment of SuM connections within the hippocampus. Of note, although the SuM is not known to express MRs at any age, DG granule neurons do express MRs, albeit at lower levels than CA2 neurons [57, 58]. Although we interpret the vGluT2 staining to reflect SuM axons, several other extrahippocampal inputs to CA2 express vGluT2, namely a number of hypothalamic nuclei [59], which could instead, or in addition, be impacted by prenatal spiro and MR knockout. However, we note that the pattern of vGluT2 staining best matches the pattern of input from the SuM rather than input from the PVN, for example, in that it appears concentrated in the deep pyramidal cell layer in CA2 [14, 60]. In addition, transection of the fimbia/fornix, which carries SuM axons to hippocampus, results in a significant loss of vGluT2 stain in CA2 [25]. For these reasons, we think that input from the SuM is impacted, but certainly vGluT2 staining may be reflecting inputs from elsewhere.

In behavioral assessments, we found that spiro-treated animals were hyper-reactive to novel objects, a contextual novelty phenotype, although they showed no deficits in a social discrimination assay. The presence of a contextual novelty phenotype, absent of a social novelty impairment, was somewhat surprising given our previous finding that whole-brain as well as CA2-targeted MR deletion impaired behaviors in response to both contextual and social novelty. Thus, embryonic MR does not appear to be sufficient to impact social behavior, which could be related to the adult gene expression. However, Chen et al. [14] reported that although inhibition of the SuM-DG pathway impairs contextual novelty detection, inhibition of the SuM-CA2 pathway does not impair social novelt y detection, suggesting redundancy in the circuit or compensatory changes in their studies and ours.

Despite prenatal exposure to spiro impairing establishment of CA2 input and output circuitry, we found no difference in adult expression levels of CA2 molecular markers, including MR, PCP4 and NECAB2. Most of the prominent histological markers for CA2 are first observed in early postnatal life, around the second postnatal week [21–23] and spiro was likely eliminated from the tissue by that age. Inhibition of MRs through spiro metabolites could also be accompanied by a subsequent physiological negative feedback response and restoration of baseline expression [16, 61]. That PCP4 and NECAB2 expression in adults are unchanged with spiro treatment is likely reflective of the normal MR levels in CA2, arguing that CA2’s transcriptional program can be recovered upon restoration of MR function.

We cannot rule out whole-body effects of treatment and the potential that spiro is also acting on MRs outside of the hippocampus such as in either the dams’ or the embryos’ peripheral nervous systems or kidneys, thus making interpretation of its site of action difficult to disentangle [62]. As yet though, we know of no better method of inhibiting MRs *in utero*, in a time-limited way, thus making it the best approach available for addressing the role of MR activity on CA2 development. We note that full MR knockout animals were not shown to develop health issues until postnatal day 5, at which time they showed weight loss and death [63], suggesting that embryonic MRs, central or peripheral, are not required for embryonic development. We also observed no indication of poor pregnancy outcomes in the dams. Additionally, as our results closely parallel the findings observed in whole brain and forebrain-specific embryonic conditional knockout models [16, 18], we think that the phenotypes observed are related to prenatal MR inhibition in the brain.

To comprehensively investigate the involvement of embryonic MR expression in the development of CA2 circuitry and function, future studies assessing the roles of CA2’s extrahippocampal projections and inputs in other CA2-dependent behaviors will be required. For example, both activation of a projection from CA2 to the lateral septum and the vasopressinergic input from the hypothalamus into CA2, are required for some social aggressive behaviors [11, 64]. Therefore, social aggressive behavior in mice may also be susceptible to prenatal exposure to spiro, possibly by disruption of the CA2 projection to lateral septum. We hypothesize that MR activity during development is required for normal connectivity, and thus normal behavior. How both are derived from the capacity of MRs to bind DNA and exert transcriptional control awaits further investigation.

## Supplementary information

Supplemental Table

## Data Availability

The datasets generated during and/or analyzed during the current study are available from the corresponding author on reasonable request.
