# Supplementary material for: Prenatal exposure to the mineralocorticoid receptor antagonist spironolactone disrupts hippocampal area CA2 connectivity and alters behavior in mice
Source: Neuropsychopharmacology. 2024 Sep 5;50(2):378–87. doi: 10.1038/s41386-024-01971-7 (PMC11631951; doi:10.1038/s41386-024-01971-7)
Supplement: Supplementary file 1 — Supplemental Table [file 41386_2024_1971_MOESM1_ESM.pdf]

Antibody	Vendor	Catalog Number	Dilution
GFP	Invitrogen	A10262	1:1,000
PCP4	Invitrogen	PA5-52209	1:500
vGlutT2	Synaptic Systems	AB2251-I	1:10,000
MR	Millipore	MABS496	1:100
NECAB2	Novus	Nbp1-84002	1:500
Goat anti-Guinea Pig IgG (H+L) Highly Cross-Adsorbed Secondary Antibody, Alexa Fluor™ 568	Invitrogen	A11075	1:500
Goat anti-Mouse IgG (H+L) Highly Cross-Adsorbed Secondary Antibody, Alexa Fluor™ 568	Invitrogen	A11031	1:500
Goat anti-Rabbit IgG (H+L) Highly Cross-Adsorbed Secondary Antibody, Alexa Fluor™ 633	Invitrogen	A21071	1:500
Goat anti-Rabbit IgG (H+L) Cross-Adsorbed Secondary Antibody, Alexa Fluor™ 568	Invitrogen	A11011	1:500
Goat anti-Chicken IgY (H+L) Secondary Antibody, Alexa Fluor™ 488	Invitrogen	A11039	1:500
